# A Review of Eating Disorders and Suicide Risk in Adolescence

**DOI:** 10.1100/tsw.2005.101

**Published:** 2005-09-28

**Authors:** Ida F. Dancyger, Victor M. Fornari

**Affiliations:** Department of Psychiatry, North Shore University Hospital, New York University School of Medicine, New York, USA

**Keywords:** suicide, suicidal behavior, risk factors, eating disorders, adolescence, United States

## Abstract

This review examines the literature during the past 10 years about suicide risk and suicide during adolescence and young adulthood of individuals with eating disorders. Epidemiological surveys are summarized, including suicide rates, parasuicidal behaviors, associated risk factors, and comorbid psychopathology. Critical implications for the comprehensive assessment and treatment planning, including safety considerations, are discussed. Two clinical cases of women with long-standing eating disorders are described to highlight both the pragmatic considerations and the complex clinical challenges of working with patients with eating disorders who become suicidal. The potentially life-threatening issues of safety have not received sufficient attention, neither in the medical literature nor by the treating clinicians. All health care professionals who are treating patients with an eating disorder must be keenly aware of the serious risks of suicidal behavior and of suicide in this population.

## INTRODUCTION

In this paper, we review the literature regarding the relationship between eating disorders (EDs) in adolescence and suicide (both attempters and completers). Several investigators have suggested that suicide is the major cause of death among patients with Anorexia Nervosa (AN), refuting the assumption that inanition generally threatens the life of these patients[1]. Two clinical vignettes will be presented that highlight the issues of suicidality during the course of the illness and its treatment. The first case example will be of a woman who developed AN while at boarding school and managed to avoid weight restoration, maintaining a body mass index (BMI) below 15 for more than 20 years despite multiple hospitalizations, and who made a life-threatening suicide attempt while in treatment. The second example will be of a woman who struggled with an ED for over 30 years and ultimately committed suicide at age 55 following a period of major depression coinciding with severe marital discord. The implications for assessment and treatment of individuals with an ED who have a history of suicidal ideation, a serious suicide plan, and/or a history of suicide attempts, require special consideration. The prevention of suicide in adolescents with an ED remains a major focus of clinical care, often underestimated by the clinician treating the individual patient and underreported in the literature.

## EPIDEMIOLOGY

Most studies regarding the mortality rate (including death by suicide) in subjects with EDs have varied in a number of important ways. Different researchers have reported a wide range of findings. This variability in outcome can be understood by examining the particular characteristics of each study sample. As in other psychiatric epidemiological surveys, the methodological variables, such as age of onset of the ED, age of subjects at time of study, comorbid psychopathology, and length of follow-up, have all contributed to different findings. As can be seen in Table 1 (organized beginning with the studies with the greatest number of subjects), a few of the investigators have reported results grouping together a number of studies incorporated into a meta-analysis; there are more studies of AN than the other EDs, and more studies with females than males. It is important to note that these studies consisted of a wide range of populations, including inpatient, outpatient, and some of the deaths had no history of hospital admission[2].

**TABLE 1**
**Studies of Suicide and Eating Disorders in Adolescence and Young Adulthood**

| Authors | Subjects: Diagnosis, Age in Years | Suicide Rates | Findings |
|---|---|---|---|
| Sullivan (1995) | 3,006 females: AN, age range = 15–24 | 27% of the deaths were due to suicide | The aggregate mortality rate was found to be much higher than the annual death rate due to all causes of death for females aged 15–24 years. |
| Emborg (1999) | 2,526 females, 237 males: AN or AN-related ED, mean age admitted to psychiatric hospital = 23.2, mean age admitted to somatic hospital = 21.3 | AN 2.2% | Patients admitted to a psychiatric hospital are more likely to commit suicide than patients admitted to a somatic hospital, who usually die of natural causes. |
| Pompili et al. (2004) | 1,536 patients: AN, age range = 14–25 | 2.3% | Suicide among patients with AN is more frequent when compared to the general population. |
| Harris and Barraclough (1997) | 1,300 females: AN BN, mean age = 20 | AN and BN 2.2% | Nearly all cases were referrals to medical or psychiatric departments. The suicide risk was 23 times the expected. |
| Keel et al. (2003) | 246 females: AN BN, age range = 12–18 | AN 4.1%<br>BN 0.4% | The best predictor of fatality was the severity of alcohol use disorder. Standardized mortality ratios were elevated for causes of mortality and suicide in AN, but not for death in BN. |
| Crisp et al. (1992) | St. George Cohort: 81 females: AN, mean age = 16.8; Aberdeen Cohort: 63 females: AN, mean age = 19.1 | St. George, 1%; Aberdeen, 6% | Complications of AN and suicide are the two most common causes of death for this disorder. |
| Powers et al. (1994) | 15 females: ED, mean age = 25.6; 15 males: ED, mean age = 25.8 | Females 0%<br>Males 6.7% | Clinical findings at the onset of treatment were very similar between males and females in terms of ED symptoms. |

*Note*: AN = Anorexia Nervosa; BN = Bulimia Nervosa; ED = Eating Disorder.

It is critical to note that since the focus of this paper is on adolescence, we have omitted studies that did not indicate a link to adolescent years in some way, either by reporting the age of onset of the subjects or the age at the time of the research was not during adolescent years. We have defined adolescence here as persons aged 10–25 years (as per a position statement of the Society for Adolescent Medicine)[3]. With this more limited review, the following conclusions can be made: the suicide rates reported for patients specifically with AN range from 2.2%[2,4] to a high of 15%[5], rates for patients with Bulimia Nervosa (BN) are reported to be 0.4%[6] to 2.2%[4], suicide rates among patients with EDs are higher for males (6.7%) than females[7]. The range of years at follow-up was from 5–23 years[1], 1.7–33 years[8], a mean of 22 years for the St. George cohort, and 21.8 years for the Aberdeen cohort[5]. The Sullivan[8] study had the largest number of subjects, as it was a meta-analysis of 42 other studies and reported that 27% of the subjects, who had died at a median follow-up of 7.6 years, had committed suicide. Other findings indicated a difference in rates of suicide between patients hospitalized in psychiatric facility (higher rates) compared to a medical facility. Overall, what is consistent is that the rates of mortality and suicide are significantly higher than the general population.

As seen in Table 2 (also listed in order beginning with the highest number of subjects), the rates of parasuicidal behavior, which includes suicide attempts, self-injurious behavior, and deliberate self-harm (cutting, burning, biting, hitting, choking) and intense suicidal ideation, differ across studies. Males with EDs exhibited more than double the attempted suicides than females and were also more frequently homosexual in their orientation[9]. Similarly, as can be seen in Table 1, for completed suicides, males were also considerably higher than females[7].

**TABLE 2**
**Studies of Parasuicide and Eating Disorders in Adolescence and Young Adulthood**

| Authors | Subjects: Diagnosis, Age in Years | Parasuicidal Behavior | Parasuicidal Rates | Associated Risk Factors | Findings |
|---|---|---|---|---|---|
| Miotto et al. (2003) | 1,000 adolescents: AN BN EDNOS, age range = 15–19 | Intense suicidal ideation | Males 5%<br>Females 5% | Depression, anxiety, obsession-compulsion, hostility, paranoid ideation, interpersonal sensitivity | Both males and females reporting suicidal ideation achieved significantly higher scores on the ED inventories. |
| Johnson et al. (2002) | 717 adolescents: AN BN EDNOS, mean age = 13.8 | Attempted suicide | With ED 12.5% | Depression, anxiety, substance use | EDs during adolescence may be associated with an elevated risk for a broad range of physical/ mental health problems during early adulthood. |
| Favaro and Santonastaso (1996) | 398 women: AN BN, age range = 20–27 | Attempted suicide | AN, BN-NP 2.7%, 5.7%;<br>AN, BN-P 20.0%, 14.1%;<br>AN, BN laxatives 18.75%, 16.7% | Obsession-compulsion, depression, anxiety, hostility, phobic anxiety | Psychiatric symptoms were significantly more serious in purging compared to nonpurging groups. |
| Corcos et al. (2002) | 295 females: AN BN, mean age = 25.3 | Attempted suicide | 1st attempt before BN 15.9%;<br>After onset of BN 59.8% | Depression, impulsive disordered conducts | BN who had attempted suicide reported more often suicidal ideation during adolescence. The suicidal group reported more often life events, such as separation from their |

| | | | | | |
|---|---|---|---|---|---|
| | | | | | family or separation in their family prior or concomitant to the onset of their ED. |
| Milos et al. (2004) | 288 females: AN BN EDNOS, age range 17–30 | Attempted suicide | AN-R 10.5%<br>AN-BP 34.7%<br>BN-P 29.9%<br>BN-NP 14.3% | Severe depression, vomiting, use of laxatives, fear of weight gain | Subjects with a purging type ED more frequently had a history of attempted suicide than subjects with a nonpurging type ED. |
| Franko et al. (2004) | 246 women: AN BN, mean age = 24.8 | Attempted suicide | AN 22.1%<br>BN 10.9% | AN severity of depression;<br>BN history of laxative abuse | Significantly more AN subjects than BN subjects made a suicide attempt. |
| Youssef et al. (2004) | 152 females: AN BN, age range = 18–24 | Attempted suicide | AN 29.7%<br>BN 30.0% | Depression, antisocial practices, hysteria, psychopathic deviate, obsessiveness, anger/fears | Suicide attempts were most frequent in subjects with purging behavior. Those attempting suicide with EDs were mostly students. |
| Welch and Fairburn (1996) | 102 women: BN, age range = 16–35 | Deliberate self-harm | BN 30% | Overdose, cutting, burning, alcohol/drug abuse | BN cases used more drugs than control groups. BN cases also had a higher rate of deliberate self-harm than controls. |
| Favaro et al. (2004) | 74 females: AN, mean age = 23.6 | Attempted suicide, impulsive self-injurious behavior, suicidal ideation | AN 82.4% reported suicide attempts, self-injurious behavior, or suicidal Ideation | Significantly lower cholesterol levels | Subjects who reported previous suicide attempts, impulsive self-injurious behavior, or current suicidal ideation showed significantly lower cholesterol levels than subjects without suicidality. |
| Bramon-Bosch et al. (2000) | 30 males, 30 females: AN BN EDNOS, mean age = 23.2, 18 | Attempted suicide | Males 50%<br>Females 23% | Anxiety and depressive disorders | Suicidal behavior was more frequent among male patients. More homosexuality in males. |
| Wildman et al. (2004) | 54 females: AN BN EDNOS, age range = 23–37 | Attempted suicide, cutting, burning, hitting, biting, choking | AN-R 18.5%<br>AN-BP 29.6%<br>BN 55.6% | Depression, anxiety, substance use | Onset of major depression occurred more often before the onset of an ED. |
| Bryant-Waugh et al. (1996) | 22 children (16 female, 6 male): AN, mean age = 3.1 | Suicidal behavior | AN 13.6% | Suicidal ideation | Clinical features of early onset AN were found with the majority of children presenting fear of fatness, distorted body image, and excessive exercising. |

*Note*: AN = Anorexia Nervosa; AN-BP = Binge Purge Anorexia Nervosa; AN-R = Restricting Anorexia Nervosa; BN = Bulmia Nervosa; BN-P= Purging Bulimia Nervosa; BN-NP = Non-purging Bulimia Nervosa; ED = Eating Disorder; EDNOS = Eating Disorder Not Otherwise Specified.

When a specific ED subcategory was indicated, generally, AN subjects made significantly more suicide attempts than BN subjects[10]. This, too, is confirmed by actual suicides being higher in AN than BN as indicated in Table 1 in one report[6] and being the same in another[4]. The latter study points out that BN patients are usually underrepresented in inpatient surveys, as most commonly they are treated in outpatient settings. Although, other investigators did not find these differences, but found that subjects with purging behavior (whether within AN or BN) made more suicide attempts[11,12]. However, when suicide attempts and self-injurious behaviors were grouped together, BN patients showed almost double the parasuicidal gestures. Although most of the studies are clinical samples, Welch and Fairburn[13] used a community sample and showed that BN subjects had higher rates of deliberate self-harm than both normal controls and psychiatric controls. At this time, there is inconsistency regarding ED subgroup diagnosis and relationship to suicidal behaviors and completed suicides.

Table 2 also highlights and summarizes some of the associated risk factors that have been identified with these parasuicidal behaviors in subjects with EDs[14,15,16,17,18,19,20]. The range of contributing variables includes severity of depression, history of laxative abuse, anxiety, obsessive-compulsive behaviors, hostility, paranoid ideation, alcohol/drug abuse, antisocial behaviors, and low cholesterol levels.

Does having experienced the traumatic event of sexual abuse contribute to the development of an ED? A recent review[21] of the relationship between traumatic experiences and the development of EDs confirmed childhood sexual abuse to be a significant, but nonspecific, risk factor especially for the development of BN in adults and adolescents of both sexes. Of particular interest was one study that examined adolescents who experienced date violence, including date rape, and found that for both girls and boys, they were more likely to have suicidal thoughts or attempts than their nonabused peers[22]. However, Brewerton[21] concludes that the links between EDs and trauma-related comorbidity, such as PTSD, remain relatively unresearched in terms of long-term course, prognosis, and treatment response.

## COMORBID PSYCHOPATHOLOGY

To care for individuals with an ED, it is essential to understand what coexisting psychiatric disorders are present in order to plan treatment effectively. Rarely do EDs occur as the sole form of psychopathology[23]. Depressive and anxiety disorders as well as substance abuse are frequently encountered. During the past 20 years, considerable attention has been given to the relationship between ED and affective disturbances. A number of investigators have documented that bulimic and anorectic subjects evidence high levels of both depression and anxiety. Several studies strongly suggest that women with AN-BP subtype are the most pathologic subgroup, experiencing more depressive symptoms than other ED subgroups. The psychiatric Axis I disorders most commonly occurring with AN and BN are depression, anxiety disorders, and substance abuse[24].

Clinicians working with an ED population have recognized the association between ED and depressive disorders for a long time. The nature of the relationship is complex and must be understood in the context of the psychological effects of starvation. The work of Keys and colleagues[25], with the Minnesota Experiment, documented that malnutrition and emaciation can produce symptoms that can be indistinguishable from depression.

Depression in an anorectic individual, however, is not always the result of malnutrition. In some instances, the mood disorder is premorbid to the ED. Data have been published on the prevalence of depression in anorectic patients who are no longer malnourished. These studies support a much higher than normal rate of depression cooccurring with AN regardless of nutritional status. In one study of psychiatric comorbidity of depression and ED, the lifetime rate of major depression was approximately 50% for the anorectic and bulimic individuals, but nearly 90% for those with AN-BP subtype[23].

Attempts to clarify the link between ED and anxiety disorders have centered on studying related traits in patients afflicted with these illnesses or in determining the frequency with which patients meet criteria for both illnesses. The high prevalence of elevated subjective and objective ratings of anxiety-related symptoms in ED individuals raises some questions as to whether or not depressive symptoms are, in fact, more frequent than anxiety-related symptoms. Fornari and colleagues[23] found that depressive disorders were not significantly more frequently encountered than anxiety disorders in an ED sample. In the same sample, a comparison was made between the various ED subgroups regarding the cooccurrence of an anxiety disorder. No significant subgroup differences were found with the exception of the AN-BP subtype being significantly more likely to have a lifetime history of obsessive-compulsive disorder than the AN-R subgroup.

Over the past 25 years, a significant body of data has accumulated, suggesting that ED and substance abuse, especially alcoholism, are interrelated. The evidence for this association is particularly compelling in individuals with BN, in which a clear increase in the rates of substance abuse in both patients and their first-degree relatives has been demonstrated[24].

A number of considerations may contribute to the fact that nearly one-quarter of all ED patients give a current or past history of substance abuse. Because many ED patients may have borderline personality features, they may be more vulnerable to drug and alcohol abuse. Others may abuse amphetamines in their drive for thinness. Some become dependent on benzodiazepines in order to fall asleep. In one study, more than half of the female patients on a substance abuse unit in a psychiatric hospital met DSM IV criteria for an ED.

As reported, EDs are associated with high rates of comorbid psychopathology including mood disorders, anxiety disorders, substance abuse, and personality disorders[23,26,27] and, in turn, these frequently present illnesses have been associated with both suicidal and parasuicidal behaviors, as well as suicide.

Numerous abnormalities in the serotonergic system in suicide completers and attempters have been described[28]). Recent evidence suggests that serotonin neuronal systems may create vulnerabilities that may make individuals susceptible to an ED[29]. Further work may help to clarify the neurobiological factors of the relationship suicide, suicidal behaviors and EDs.

## TREATMENT IMPLICATIONS

As discussed in Dancyger and colleagues[30], ED patients with serious comorbid psychiatric conditions (especially suicidal ideation or intractable depression) may not only require admission to an inpatient facility, but may also be extremely challenging for mental health practitioners. As outlined in the APA Practice Guidelines[31] that are in agreement with the authors' own clinical experiences, it is imperative to evaluate and assess every patient who presents with AN, BN or EDNOS thoroughly, with a comprehensive psychiatric interview, especially reviewing history of suicidal ideation, parasuicidal behavior, suicidal attempts, and family history of suicidal behavior and suicide. During the assessment phase, particularly with children who may be quiet during the family assessment, it is imperative to talk in private with the child and question directly for any possible suicidal thoughts and any history of abuse[32]. Also critical to consider are the ethical and legal issues surrounding treatment compliance and refusal, by either the child, the adolescent, or the family of the individual with an ED. The need for the mental health practitioners to continue outpatient treatment or to hospitalize even against the patient's or the family's wishes when lives are at risk[31] remains a therapeutic challenge for the treating clinicians. Understanding the family history of psychopathology and suicidal behavior is often critical to the proper clinical care of the adolescent with an ED.

## CLINICAL VIGNETTES

### Case 1

Ms. A was a 16-year-old student at a boarding school who had become notably thinner during her junior year in high school. Her parents reportedly advised her to increase her food intake, thinking that this would be an adequate remedy to her "skinny" state. Although she was unable to regain the weight, Ms. A received so many favorable comments about her appearance from her friends that she enjoyed the attention. During a summer family vacation, her parents noticed that she hardly ate. The parents sought consultation in order for their daughter to get a "proper meal plan" for weight restoration.

Psychiatric evaluation revealed that the family had endured multiple stressors including the suicide of an older cousin at the age of 21. The family had financial problems coupled with severe marital discord between the parents. The parents seemed puzzled when the therapist suggested that these issues might be in some way related to Ms. A's eating difficulties.

Ms. A entered an outpatient treatment program aimed at weight restoration. When Ms. A failed to make the required weight goals (after proximately what period of time?), she was referred for an inpatient hospitalization at an Adolescent Medicine Inpatient Unit in a general hospital in suburban New York. Because weight gain was not sufficient, Ms. A was subsequently transferred to a specialized Eating Disorder Inpatient Unit in a psychiatric hospital. Weight gain remained modest, not reaching her goal for discharge.

On discharge, Ms. A was treated in a day treatment program specializing in ED, where the balance of the weight restoration was to be accomplished. Suicidal thoughts became a focus of clinical concern as she began to recall traumatic childhood memories of sexual abuse (that she did not know whether they were real or imagined). In group therapy, Ms. A befriended a young woman with a history of multiple suicide attempts. Unbeknownst to the therapist or the other group members, Ms. A entered into a suicide pact with her fellow patient. The two planned to hoard their medications, drink what they thought would be a sufficient quantity of alcohol, and left suicide notes for their families. After swallowing the pills and drinking the alcohol, Ms. A called her therapist and left a message "in order to say goodbye". On receiving the message, the therapist contacted the local police department. Police officers went to her apartment, forcibly opened the door, and found the two young women alive, but unconscious. The two young women were brought to the ER and then treated in an intensive care unit until they were medically stable. Once stable, they were transferred to separate locked psychiatric units specializing in ED. Ms. A was angry to have been rescued and remained actively suicidal for several weeks. Her family was upset with the treatment team for insisting that she be treated against her and their wishes. It was suggested to the family that they seemed more interested in the protection of free will than her survival. The therapist speculated why they might unconsciously wish her dead. Did Ms. A have the knowledge of a family secret? Was the family more willing to allow her to die than face the consequence of disclosure?

The family struggled with the treatment team and wondered why "nothing was being done to help!" After a long period of confrontation and supportive measures, Ms. A developed a trusting relationship with her therapist. At follow-up, Ms. A has remained at a chronically low weight, but has managed to remain outside of a hospital for over 10 years. Despite having a BMI of 16, Ms. A reports that she has regular menses. Although Ms. A acknowledges that she often feels that "life is not worth living", there have not been further suicide attempts reported to her therapist.

### Case 2

Although Ms. N had struggled with her moods throughout her childhood and adolescence, it was not until she had lost a significant amount of weight as a 17 year old that she discovered the power of regulating her weight. The only daughter in a working class family, Ms. N had always been ashamed of her mother's leg deformities that resulted from polio. In college, Ms. N excelled and she decided to become a pharmacist. Ms. N married in her early 20s and reported that her weight would fluctuate dramatically by 30–40 lbs. Ms. N maintained a wardrobe in three sizes depending on her weight. Her husband wondered why such a variation, but no formal consultation was obtained. After the birth of her second child, Ms. N learned that her mother had committed suicide with an overdose of medication she had been hoarding. Although devastated by this tragic loss, Ms. N intellectualized how difficult walking had been for her mother and that "she was no longer in distress". Ms. N continued to have dramatic weight fluctuations including periods when her weight fell so low that she did not menstruate for 6 months. Finally, at the insistence of her husband, Ms. N was sent for consultation. When confronted with her ED, Ms. N impulsively took an overdose of her own medication that she had in the medicine chest and was hospitalized, first medically and then in a psychiatric unit. The couple argued and decided to separate. Ms. N felt resolved that this would allow her to be in charge of herself. Her moods fluctuated as dramatically as her weight. Subsequently, Ms. N and her husband divorced. The weight fluctuations persisted. Mood fluctuations were also pronounced. Ms. N began outpatient psychotherapy and was prescribed a mood stabilizer to help her to minimize the dramatic shifts in her mood. Ms. N remained successful at work. Five years after her divorce, Ms. N began a new relationship with a man 10 years her senior. They moved in together. He became concerned with her mood and weight fluctuations. Ms. N discontinued her psychotherapy and medications. Following a period of significant turmoil, the two decided to separate. Ms. N was found dead by her boyfriend in their home. Ms. N had left a suicide note. In the note, Ms. N asked her family to understand that, like her mother, she too preferred not to live with the pain and humiliation. She was 55 years old at the time of her death and had had an ED for at least 36 years.

## CONCLUSIONS

It is clear that there is a strong relationship between EDs, suicidal behavior, and suicide during adolescence and young adulthood. Epidemiological surveys have confirmed the considerably high suicide rate as well as prevalent parasuicidal behaviors. As has been mentioned earlier, rarely do EDs present as the sole form of psychopathology. The associated comorbidity generally found in this population may likely be related to the increased risk of suicide.

Clinician awareness of the heightened risk of suicide in ED patients is critical during the assessment and the ongoing treatment of these individuals. Clinicians must be sensitive to monitor their own negative reactions with ED patients who are suicidal, in order to maintain a hopeful stance for recovery. As chronicity sets in, the individuals often tire, as do their families, leading to hopelessness and despair. In order to attempt to prevent the patient's death, the clinician must monitor that hopelessness in the patient and the family. It is sometimes necessary to intervene against the wishes of both the patient and even the family, who have given up hope of recovery. The legal capacity of the clinician to do so may vary from state to state and country to country, depending on the mental health laws of the region. When the clinical course reaches an impasse, obtaining outside consultation, such as a second opinion, may enhance the clinical care and support the treating clinician. It is imperative that the clinicians not contribute and unwittingly collude with the patient and the family.

This is a very complex and challenging population, and the clinician, working in a collaborative team, would be supported in order to prevent the possible demoralization of the therapist. The suicide of any patient, including an ED patient, when in treatment, is a crisis for the clinician. It is recommended that discussion in supervision, peer group, or during a team meeting may help the clinician work through this difficult clinical experience.

In summary, future research is likely to elucidate the psychobiologic underpinnings of EDs and comorbid psychopathology, which may lead to better prevention and treatment strategies for patients with EDs who have suicidal behavior.

## BIOSKETCHES

**Ida F. Dancyger, PhD,** Department of Psychiatry, North Shore University Hospital, New York University School of Medicine, New York. E-mail: IDancyge@nshs.edu

**Victor M. Fornari**, **MD**, Department of Psychiatry, North Shore University Hospital, New York University School of Medicine, New York. E-mail: VFornari@nshs.edu.
